# Heilige und die Urologie: „Für jede Krankheit den richtigen Heiligen“

**DOI:** 10.1007/s00120-021-01474-z

**Published:** 2021-02-23

**Authors:** Marie-Isabelle Schwarzburger, Friedrich H. Moll, Felicitas Söhner

**Affiliations:** 1 Institut für Geschichte, Philosophie und Ethik der Medizin, Centre for Health and Society, Heinrich-Heine-Universität, Düsseldorf, Deutschland; 2 Lehrstuhl für Mittelalterliche Geschichte, Heinrich-Heine-Universität, Düsseldorf, Deutschland; 3 Curator Museum, Bibliothek und Archiv, Deutsche Gesellschaft für Urologie e. V., Düsseldorf-Berlin, Berlin, Deutschland; 4 Urologische Klinik, Urologischer Arbeitsplatz Krankenhaus Merheim, Kliniken der Stadt Köln GmbH, Neufelder Straße 32, 51067 Köln, Deutschland

**Keywords:** Geschichte der Urologie, Medizingeschichte, Hagiographie, Hagiotherapie, Schutzheilige, Krankheitspatrone, St. Liborius, St. Rochus, St. Apollinaris, St. Vitus, History of urology, History of medicine, Patron saints, Saint of diseases, Hagiotherapy, St. Libory, St. Roche, St. Apollinaire, St. Vitus

## Abstract

Zu den diversen frühneuzeitlichen Heilungsangeboten gehörte auch die Hagiotherapie, die für den Bereich der Urologie, insbesondere bei Harnsteinleiden oder den seuchenartig auftretenden Geschlechtskrankheiten, in den verschiedenen deutschen Regionen eine wichtige Rolle einnahm. Noch heute werden besonders in katholischen Gegenden weltweit der Hl. Liborius oder der Hl. Rochus neben dem Hl. Apollinaris oder dem Hl. Dionysius durchaus erinnert.

## Abstract (en)

During Medieval and Renaissance times up to the 19th century hagiotherapy was a common part of many different health offerings in society. Within the field of urology, kidney stone disease and venereal (sexually transmitted) diseases were the favourite subjects. Even today, the names of St. Libory, St. Roche, St. Apollinaire and St. Dionysius are common within the culture of remembrance in Europe and the USA.

## Einführung

Die Untersuchungen zur Urologie- und Medizingeschichte haben sich hin zu einer Kulturgeschichte des Medikalen geweitet. Untersucht wurden und werden von „clinician historians“ meist Krankheitsphänomene und häufig technische Aspekte und Entwicklungen des medizinischen Querschnittsfachs. Doch haben sich heute Perspektiven verändert. So ist heute das Forschungsinteresse auch auf Einstellungen und Praktiken, die mit Gesundheit und Krankheit (hier mit Erkrankungen des Harntraktes sowie der [männlichen] Sexualsphäre) zu tun haben, fokussiert. Auch der Prozess der „Medikalisierung“ der Sphäre von Harntrakt und Genitalorganen rückt in das Interesse von gender- und körpergeschichtlichen Aspekten.

Für solche Aspekte ist ein epochenübergreifender, häufig lokalhistorischer Ansatz besonders ergiebig. Umgekehrt weitet eine Kulturgeschichte des Medikalen auch den Horizont für neue regionalhistorische Fragen, etwa danach, ob oder wie „medikale Regionen“ entstanden und entstehen und wie sie sich mit anderen vergleichen lassen [1–4].

In der fachkulturellen Erinnerung der Urologie sind beim Harnsteinleiden neben der Heiligenvita des Hl. Liborius[5, 6] im Rheinland die des Hl. Apollinaris oder die des Hl. Rasso von Grafrath (Andechs; [7]) in Bayern bekannt, manchmal wird noch der Hl. Benedikt [8] zitiert.

Für den Erkrankungskomplex der Geschlechtskrankheiten („Widerfahrnis“) sind zumeist der Hl. Fiacrius, der Hl. Dionys(ius), der Hl. Georg, der Hl. Pellegrinus, der Hl. Vitalis von Assisi, im Rheinland wieder der Hl. Apollinaris von Ravenna oder der Hl. Rochus von Montpellier in Tradition und als Stationsbezeichnung [9] in Krankenhäusern aktiv in Gebrauch [10, 11], wobei der Hl. Rochus wohl eher in seiner Funktion als Pestheiliger (Seuchenheilige, zu denen auch die Geschlechtskrankheiten gerechnet werden) als Namensgeber herangezogen wird. Der Hl. Vitus, einer der 14 Nothelfer, wird im Rheinland (Köln) bei Inkontinenz angerufen [6] Bei den Heiligen wechseln die Zuschreibungen der Erkrankungen (Syphilis, Geschlechtskrankheiten; Nieren‑/Gallensteine etc.) häufiger [12–16]. In anderen medizinischen Fachgebieten wie z. B. der Ophthalmologie (Hl. Lucia von Syrakus, Hl. Odilia/Ottilie) oder Zahnheilkunde (Apollonia von Alexandria) haben sich ebenfalls ein Traditionsbezug auf Heilige in der Erinnerungskultur der Fächer und der Bevölkerung erhalten [17–20].

Der Schutzpatron ist nicht nur in der katholischen Kirche ein Heiliger, der bei unterschiedlichen Wünschen um Beistand gebeten wird [21]. Die den Patronen zugeschriebene Heilkraft konnte an allen Orten erbeten werden, dennoch kristallisierten sich einzelne Orte heraus, an denen die Schutzheiligen besonders verehrt wurden. Zumeist waren dies Stätten, an denen sich Reliquien der Heiligen befanden [22].

Wir wollen der Frage nachgehen, wie sich in zwei verschiedenen Kulturräumen (im Rheinland sowie in Schwaben) anhand von Patrozinien Heilige mit Bezug zur Urologie nachweisen lassen und ggf. ein besonderer Kultus vorliegt. Dies wollen wir mit zeitentsprechenden Zeugnissen (u. a. Votive) für frühe „urologische Tätigkeit“ in Verbindung setzen. Weiterhin wollen wir fragen, inwieweit sich dies in der lokalen spezifischen Ikonographie niederschlägt [23].

Für das Rheinland^4^ lassen sich wesentliche Patrozinien nachweisen: für den Hl. Liborius, Schwerpunkt Paderborn [24] u. a. in Wetter-Wengern, für den Hl. Rochus u. a. in Köln-Bickendorf, Rochuskapelle an der Venloer Straße, Kölns sowie in Bergisch-Gladbach Hand (erbaut 1667–1668), in Düsseldorf-Pempelfort, in Bonn-Duisdorf; für den Hl. Apollinaris in Düsseldorf Oberbilk, in Lindlar-Fielingsdorf, in Wermelskirchen und in Remagen (Wallfahrtskirche).

Die Verehrung des Hl. Liborius reichte bis in den süddeutschen Raum hinein [25]. So finden wir Patrozinien des Hl. Liborius in Oberotterbach bei Rottenburg a.d.L. (Niederbayern) und in Bamberg. Das dortige Domstift stand in enger Verbindung mit der Paderborner Kirche [26]. Auch fand der Hl. Liborius Einzug in die Heiligenlitanei des bayerischen Klosters Tegernsee^1^ [25, 27]. Die Benediktinermönche in Tegernsee riefen Liborius mit „Sancte Libori ora pro nobis“ zwischen den Heiligen Julius und Maxentius an [25].

Für den Hl. Rochus finden sich in Bayern weitere Patrozinien beispielsweise in Bad Kohlgrub (Oberbayern), Egg an der Günz (Schwaben), Gaishard (Schwaben), Landshut (Niederbayern), Lohr am Main (Unterfranken), Nürnberg (Mittelfranken), Obersteinbach (Mittelfranken), Schwabmühlhausen (Schwaben), Waltenhofen (Schwaben) sowie in Zirndorf (Mittelfranken).

Nachfolgend sind die bekannten Orte zu den Verehrungen der Heiligen aufgelistet. Die Liste erhebt keinen Anspruch auf Vollständigkeit (Tab. 1).

**Table 1:** 

Liborius von Le Mans		Rochus von Montpellier		Apollinaris von Ravenna		Dionysius von Paris	
Rheinland/Westfalen	Bayern	Rheinland	Bayern	Rheinland	Bayern	Rheinland	Bayern
Paderborn	Rothenburg o.d.T.	Köln	Bad Kohlgrub	Aachen	–	Duisburg-Serm	Regensburg
Essen	Tegernsee	Aachen	Bruckmühl	Burtscheid	–	Düsseldorf	Vierzehnheiligen (Bad Staffelstein)
Bonn	Bamberg	Seligenthal (Siegburg)	Egg an der Günz	Düsseldorf	–	Krefeld	Kaufbeuren
Trier	Oberotterbach	Bingen am Rhein	Gaishardt-Bissingen	Abtei Siegburg	–	Köln-Longerich	Waal-Schwaben
Mainz	–	Bonn	Landshut	Remagen	–	–	Oberfahlheimnersingen
–	–	Düsseldorf	Langerringen	Köln	–	–	Pipinsried-Hilgertshausen
–	–	Mainz	Lohr am Main	–	–	–	–
–	–	–	Marktoberdorf	–	–	–	–
–	–	–	Marxheim	–	–	–	–
–	–	–	München	–	–	–	–
–	–	–	Nürnberg	–	–	–	–
–	–	–	Oberstaufen	–	–	–	–
–	–	–	Obersteinbach	–	–	–	–
–	–	–	Schwabmühlhausen	–	–	–	–
–	–	–	Raim	–	–	–	–
–	–	–	Sollach-Miesbach	–	–	–	–
–	–	–	Türkenfeld	–	–	–	–
–	–	–	Waltenhofen	–	–	–	–
–	–	–	Wessobrunn	–	–	–	–
–	–	–	Zirndorf	–	–	–	–
–	–	–	Seeg	–	–	–	–

## Heiligenviten

Einen wesentlichen Aspekt der mittelalterlichen und frühneuzeitlichen Heiligenverehrung bilden nicht zuletzt die Berichte über die Heiligenleben und Angaben über die Wundertätigkeit von Reliquien der Heiligen.

## Liborius von Le Mans

Über das Leben des Hl. Liborius ist wenig bekannt, er war 49 Jahre lang Bischof von Le Mans [28] und verstarb am 9. Juni 397 [29]. Gedenktag ist der 23. Juli [31]. Auskunft darüber liefern vier Viten des Hl. Liborius, die in der Acta Sanctorum [30] überliefert sind [31].

Bischof Aldrich (832–857) übergab im Jahre 836 die Reliquien des Hl. Liborius, der schon früh in Le Mans verehrt wurde, an den Boten des Paderborner Bischofs Badurad (815/22–862; [32, 33]). Grund hierfür war die geplante „Verbrüderung“/Verbindung der beiden Bistümer [34].

Initiator der Translation war der Kaiser Ludwig der Fromme (778–840, Kaiser 813–840), Sohn Karls des Großen (747–814, Kaiser 800–814), seine zweiten Frau Judith (795–843) und sein Sohn Karl der Kahle (823–877, Kaiser 875–878; [35]). Seit dem frühen 11. Jahrhundert wurde Liborius sowohl der Patron des Doms als auch der Stadt Paderborn [33]. In der Diözese Paderborn begann die Verehrung des Heiligen mit der Translation und dem Zug durch das Land.

Der Mainzer Erzbischof Werner von Eppstein (1225–1285) 1267 wurde bei einem Besuch der Reliquien des Hl. Liborius von einem Steinleiden befreit. Seit diesem Zeitpunkt wurde Liborius der Patron der Steinleidenden [36].

**Figure 1:**
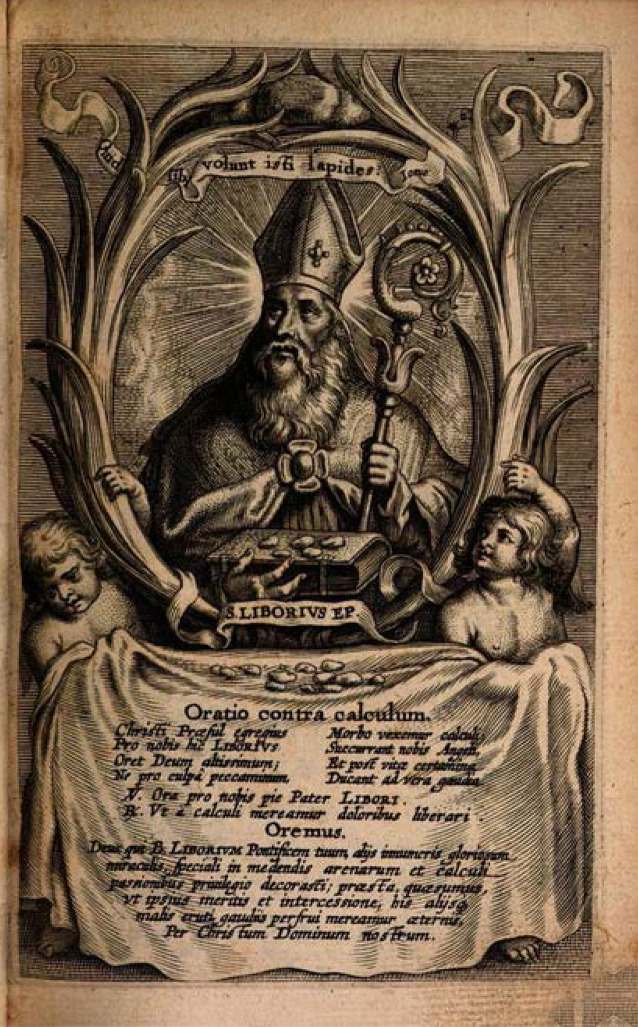

Seine Attribute sind 3 Steine. Diese dürfen nicht mit den drei Broten, die dem Hl. Nikolaus zugeordnet werden, verwechselt werden (Abb. 1).

Der rheinisch-westfälische Raum entlang des Translationsweges wurde früh von einem Libori(us)kult erfasst.

## Hl. Rochus von Montpellier

Die historische Person Rochus ist in der Forschung umstritten [37]. Laut Legende lebte er zwischen 1295 und 1379. Er widmete sich als Arzt der Versorgung von Pestkranken, die nach dem vermehrten Auftreten der Syphilis bald auch mit der „Lustseuche“ in Verbindung gebracht wurden [38].

Die Legende wurde von Francesco Diedo 1478 verfasst und berichtet, dass seine Eltern von adeligem Geschlecht in Montpellier waren und er nach ihrem Tod sein Erbe spendete. Danach pilgerte er nach Rom, wo er sich um die Pestkranken kümmerte. Bei deren Pflege erkrankte er selber an der Pest. Laut seiner Legende wird er von Engeln gesund gepflegt. Aufgrund der Verunstaltungen durch die Pest wird er entstellt. Als er in seinen Heimatort zurückkehrt, erkennt ihn dort niemand mehr und er wird der Spionage verdächtigt und zu einer Gefängnisstrafe verurteilt. Nach 5 Jahren Haft stirbt er. Durch ein Mal auf der Brust in Form eines Kreuzes, welches er schon seit seiner Kindheit hatte, kann man ihn identifizieren [37, 39].

In einigen Regionen wird der Heilige zu den 14 Nothelfern gezählt. Nicht nur Stationen für Dermatologie-Venerologie, sondern auch Krankenhäuser werden nach ihm benannt [40, 41].

Die ersten Nachweise einer Verehrung des Hl. Rochus finden sich in Italien um 1469 [42]. Durch die Überstellung der angeblichen Reliquien nach Venedig 1485 steigerte sich der Kult um den Heiligen [43]. Wie nicht anders zu erwarten war, brachten die Pestausbrüche vom 15. bis zum 18. Jahrhundert immer wieder eine lokale Verehrung der Pestheiligen auf: z. B. 1464 taucht Rochus das erste Mal bildlich auf dem Antonius-Altar des Antonio Vivarinis auf [44]. Das ist der Beginn der Verehrung in der venezianischen Malerei und sie kommt gleichzeitig mit der 1450 erneut ausgebrochenen Pestpandemie. Interessant ist, dass sowohl die Pest, als auch die Verehrung des Hl. Rochus ihren Ausgangspunkt in Venedig hatten [45]. Auch in Deutschland verbreitet sich der Kult des Hl. Rochus parallel zum Pestgeschehen. Hier ist eine erste Verehrung ab 1484 nachweisbar. In diesem Jahr erschien eine deutsche Übersetzung der Rochus-Legende [42]. So werden in Köln im Mittelalter in der Malerschule einige Gemälde von Rochus gemalt, nachdem die Pest dort ausgebrochen ist. Die Pestkapelle St. Rochus soll während der letzten großen Pest im 17. Jahrhundert entstanden sein [46]. Im Rheinland gilt Bingen am Rhein als Zentrum der Rochus-Verehrung. Aber auch Düsseldorf ist ab 1666 ein Ort der Rochus-Verehrung. In diesem Jahr erreichte die Pest den Ort am Rhein. Als Gegenwehr entschloss man sich, eine dem Rochus geweihte Kapelle zu errichten. Als Ort wurde Pempelfort gewählt, vermutlich da sich dort isolierte Häuser für Pestkranke befanden. Es ist zu beobachten, dass das Interesse an der Kapelle nach dem Ende der Pestwelle verschwand. Weitere Kapellen in Düsseldorf finden sich in Angermund und Hamm [47].

**Figure 2:**
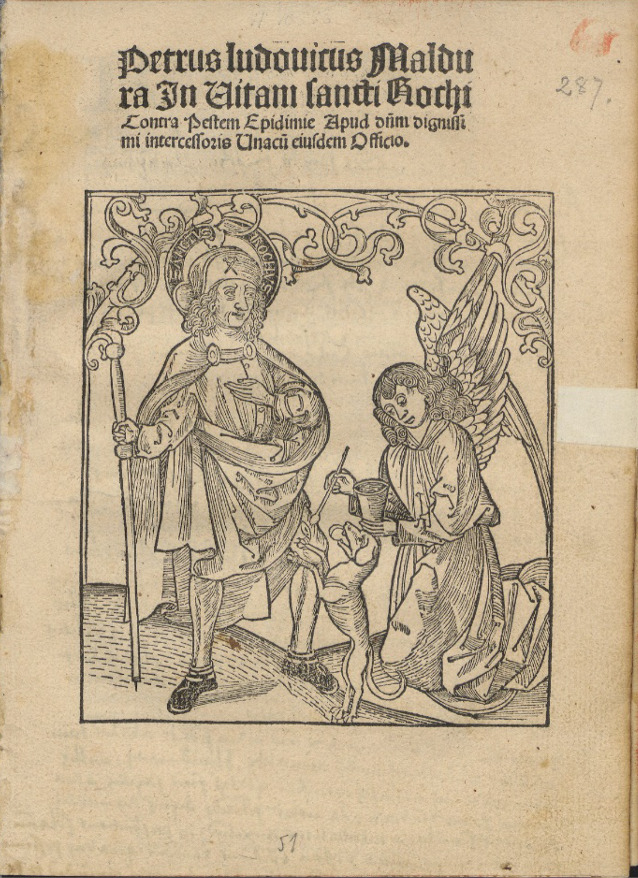

Der Rochus-Tag ist am 16. August (Abb. 2).

## Apollinaris von Ravenna/Antiochien

Der Hl. Apollinaris lebte um 200 in Ravenna [48, 49] und soll laut Legende ein Jünger des Petrus gewesen sein [48]. Er gilt als Gründer der christlichen Gemeinde von Ravenna und soll deren erster Bischof gewesen und als Märtyrer gestorben sein.

Kaiser Otto III. (980–1002, Kaiser ab 996) brachte den Kult um das Jahr 1000 nach einem Besuch in Ravenna nach Burtscheid bei Aachen^2^ für sein dort gestiftetes Apollinaris-Kloster [50]. Auch in der rheinischen Abtei Siegburg wurden Reliquien des Hl. Apollinaris verehrt. Vermutlich kamen diese aus Dijon durch Erzbischof Anno II. (1010–1075) von Köln. Von diesen beiden Orten verbreitete sich der Kult im Rheinland [49]. Nach der Auflösung der Abtei Burtscheid im 13. Jahrhundert wurden die Reliquien nach Remagen gebracht [48]. Die Propstei Apollinarisberg bei Remagen entwickelte sich seit dem 14. Jahrhundert zu einem wichtigen Wallfahrtsort [49].

Der Legende nach soll Remagen die Reliquien um 1164 durch Erzbischof Reinald von Dassel (1114–1147) erhalten haben, als dieser die Reliquien der Heiligen Drei Könige nach Köln brachte und das Schiff unversehens auf dem Rhein bei Remagen stoppte „ecce navis in medio Rheni immobilis“ [51].

Gesichert ist, die Reliquien kamen wahrscheinlich erst um 1364 nach Remagen [52]. Die Gebeine wurden vom Jülicher Herzog Wilhelm I. (1348–1408, Herzog 1380 bis 1408)^3^ 1383 nach Düsseldorf in die Stadtkirche St. Lambertus gebracht, als dieser Düsseldorf zu einem neuen Wallfahrtsort ausbauen wollte [49]. Zuvor hatte ein Ritter mit Namen Gerhard von E/i(y)nenberg (1366–1402; [53]) den Kopf „vor den Gesandten des Herzogs“ auf der Landskrone bei Bad Neuenahr um 1380–1394 versteckt, sodass später nur dieser in Remagen verblieb [54].

Besondere Orte der Verehrung sind neben Ravenna, Remagen, Düsseldorf und Köln, dort gibt es alleine acht Kirchen mit Apollinaris-Reliquien [55]. Der katholische Gedenktag des Heiligen ist der 23. Juli, in der orthodoxen Kirche fällt dieser auf den 20./23. Juli.

In Düsseldorf werden die Reliquien des Hl. Apollinaris in der St. Lambertuskirche aufbewahrt und zu jährlichen Prozessionen durch die Stadt getragen. Bis heute findet die „größte Kirmes am Rhein“ jedes Jahr um seinen Namenstag am 23. Juli statt.

**Figure 3:**
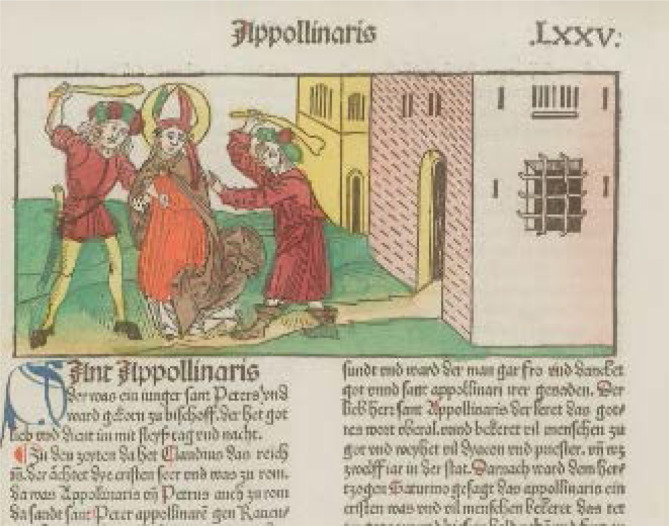

Der Hl. Apollinaris gilt als Schutzheiliger des Weines und Wassers und wird im Rheinland bei Kopfleiden (Kopfreliquie), Epilepsie, Gallen- und Nierensteinen, Geschlechtskrankheiten und Gicht angerufen ([56, 57]; Abb. 3).

## Hl. Dionys(ius) von Paris

Über das Leben des Hl. Dionysius ist kaum etwas bekannt. Er war in der zweiten Hälfte des 3. Jahrhunderts Bischof von Paris [58]. Der Hl. Dionys von Paris wird seit dem Jahr 1450 zu den 14 Nothelfern gezählt. Sein Gedenktag ist der 9. Oktober.

**Figure 4:**
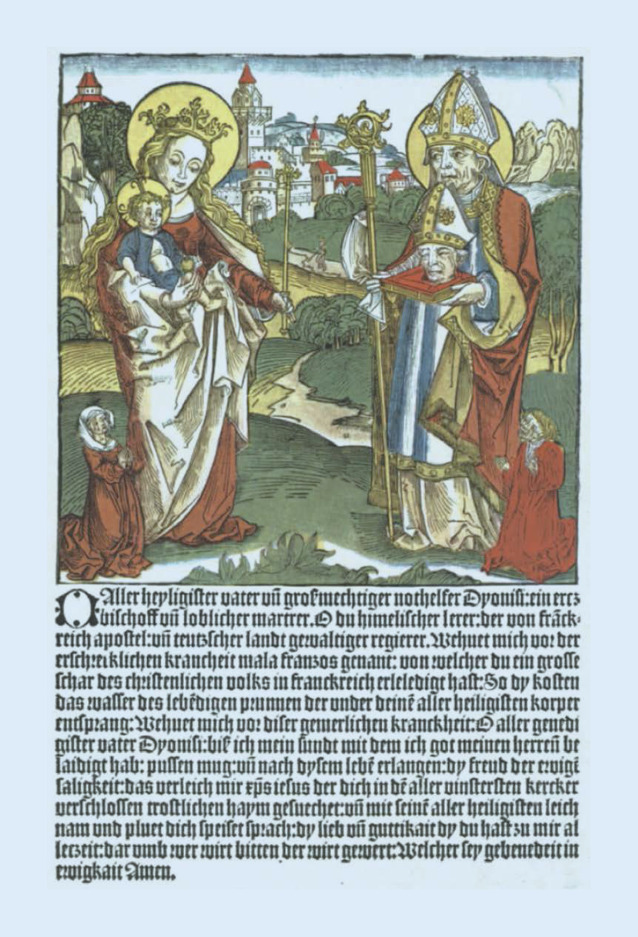

Im Rheinland ist Krefeld ein besonderer Ort der Verehrung, hier kommt er sogar im Stadtwappen vor. In einem Schöffensiegel von 1463 ist bereits der Heilige dargestellt, auf das sich die späteren beziehen. Schon in einem Flugblatt um 1495 wird der Heilige zum Schutz vor Geschlechtskrankheiten aufgeführt ([59, 60]; Abb. 4).

## Wertung

In früheren Jahrhunderten konnten Menschen aus vielfältigen Heilungsangeboten auswählen, wobei die klassisch medizinischen, der Viersäftelehre (Humoralpathologie) entstammenden wie die Diätetik, die Gabe von Drogen durch Medici puri aber auch Kräuterfrauen oder operative Eingriffe durch Lithotomisten, Bader und Wundärzte jeweils einen Teilaspekt eines Gesamtangebots bildeten. Zu diesen Angeboten gehörte auch die Hagiotherapie. Da sich Katastrophen, wie Epidemien, Hungersnöte und Unwetter, durch menschliche Macht nicht abgewendet werden konnten, suchte die Bevölkerung die Hilfe heiliger Fürbitter. Bereits in altchristlicher Zeit suchten Christen die Hilfe von Märtyrern, die in ihren Leben als Heilkundige helfen konnten [19]. Ab dem Mittelalter kristallisierten sich spezifische „Zuständigkeitsbereiche“ einzelner Patrone heraus. Dabei lassen sich durchaus regionale Unterschiede und Spezialisierungen für bestimmte Krankheitssymptome feststellen. Die Anrufung überirdische Mächte bildete einen wesentlichen Aspekt beim Umgang mit Erkrankungen [61]. Die Volksfrömmigkeit prägte nicht nur die Gesundheits- und Glaubensvorstellung, sondern nahm auch deutlichen Einfluss auf die Entwicklung der bildenden Kunst und der Volkskunst. Die Heiligen wurden als Mittler zwischen den Menschen und Gott angesehen. Sie galten als Helfer und Heiler bei verschiedenen Sorgen und Krankheiten. Die nationalen und religiösen Traditionen zeigen Unterschiede in Abhängigkeit von den lokalen und kulturellen Bedingungen. Obgleich die katholische Kirche durchaus nicht lehrte, dass Schutzpatrone Krankheiten heilen könnten, wurden Heiligen und Heiligenreliquien im Krankheitsfall eine heilbringende Wirkung zugesprochen. Die Helferqualitäten der Heiligen leiteten sich zwanglos aus deren Biographie und Martyrium ab [62]. Weiterhin spielten die Heiligen als Mittlerfiguren zwischen dem Kranken und Gott eine besondere Rolle [63–65]. Bei den Erkrankungen sind für die Urologie das Harnsteinleiden und die Geschlechtskrankheiten eine antropomorphe Konstante, was sich bis in die Hippokratischen Schriften zurückverfolgen lässt. Syphilis und Gonorrhö konnten bis zu den Untersuchungen Philippe Ricords (1800–1889) 1837 [66] nicht sicher unterschieden werden („Dualitätslehre“), spielten aber ab der frühen Neuzeit durch eine seuchenhafte Verbreitung durch den Geschlechtsverkehr und nur ungenügende Therapieoptionen durch Quecksilber (Merkuralisten) und Guaijakholz (Antimerkuralisten; [67, 68]) eine wichtige Rolle in der Gesellschaft. Da es sich bei der Syphilis um eine „neue“ Krankheit handelte, mussten Heilige, die schon vorher für Seuchen oder Hautaffektionen angerufen wurden, nun auch hier Fürbitte leisten.

Im Rheinland wie in Bayern standen Wallfahrt, Heiligenverehrungen und Votivgaben in vielfältiger Weise miteinander in Beziehung [69]. Sie stehen als Zeugnisse einer weit zurückreichenden Tradition der Volksfrömmigkeit und Hagiotherapie.

## Fazit für die Urologie

- Der Hl. Liborius ist in der Erinnerungskultur der Urologie fest verankert.
- Der Hl. Rochus, der Hl. Dionys, der Hl. Fiacrius sind bei Geschlechtskrankheiten bis heute in der Tradition. Da Geschlechtskrankheiten aber zumeist sozial negativ konnotiert waren, konnte sich zum einen kein einzelner Heiliger wesentlich durchsetzen, auch wurde bei diesen Heiligen weitere Zuschreibungen in der Tradition der Verehrung beibehalten.
- Der Hl. Apollinaris gegen Harnsteine und Geschlechtskrankheiten besitzt nur im Rheinland zwischen Remagen und Düsseldorf eine lokale Bedeutung.
- Der Hl. Vitus wird in der Region in einzelnen Nothelfergruppen mit einem kleinen Kessel – dem Marterinstrument – dargestellt, das als Urintopf/Nachttopf in der Tradition umgedeutet wurde.

## Footnotes

4 Das Rheinland, das sich historische aus vielen Territorien seit dem Mittelalter zusammensetzte und erst im 19. Jahrhundert durch die französische 1797/1815 preußische Rheinprovinz eine strengere geographische Abgrenzung erhielt, wird hier als die Region der im Dreieck Aachen – Düsseldorf – Köln/Bonn/Koblenz Region verstanden. vgl. zur Abgrenzung schon früh: Hansen J 1925 Rheinland und die Rheinländer. Kurt Schröder Bonn S 8–9 online: https://www.dilibri.de/rlb/content/pageview/1100193 Zugegriffen 20.06.2020.

1 München, Bayer. Staatbibliothek, Hs. Clm. 18121 nach Vry 1997 21

2 Otto III. hatte der Aachener Münsterkirche um diesen Zeitpunkt die Gebeine der Hl. Corona vermacht.

3 Die Zählung der Grafen von Jülich ist in der Literatur nicht einheitlich. So wird Wilhlem I. der Vater auch mitunter als Wilhelm VI. gezählt, da der Sohn und Mitregent Wilhelms IV. ebenfalls als Wilhelm V. gezählt wird. Als Markgraf (seit 1336) und als Herzog (seit 1356) wird er als Wilhelm I. gezählt.
